# Cluster Headache and Hypoxia: Breathing New Life into an Old Theory, with Novel Implications

**DOI:** 10.3390/neurolint16060123

**Published:** 2024-12-04

**Authors:** Jonathan M. Borkum

**Affiliations:** Department of Psychology, University of Maine, 301 Williams Hall, Orono, ME 04469-5742, USA; jborkum@hpmaine.com

**Keywords:** cluster headache, hypoxia inducible factor, genes, neurochemistry, pharmacology, prevention, trigeminovascular

## Abstract

Cluster headache is a severe, poorly understood disorder for which there are as yet virtually no rationally derived treatments. Here, Lee Kudrow’s 1983 theory, that cluster headache is an overly zealous response to hypoxia, is updated according to current understandings of hypoxia detection, signaling, and sensitization. It is shown that the distinctive clinical characteristics of cluster headache (circadian timing of attacks and circannual patterning of bouts, autonomic symptoms, and agitation), risk factors (cigarette smoking; male gender), triggers (alcohol; nitroglycerin), genetic findings (GWAS studies), anatomical substrate (paraventricular nucleus of the hypothalamus, solitary tract nucleus/NTS, and trigeminal nucleus caudalis), neurochemical features (elevated levels of galectin-3, nitric oxide, tyramine, and tryptamine), and responsiveness to treatments (verapamil, lithium, melatonin, prednisone, oxygen, and histamine desensitization) can all be understood in terms of hypoxic signaling. Novel treatment directions are hypothesized, including repurposing pharmacological antagonists of hypoxic signaling molecules (HIF-2; P2X3) for cluster headache, breath training, physical exercise, high-dose thiamine, carnosine, and the flavonoid kaempferol. The limits of current knowledge are described, and a program of basic and translational research is proposed.

## 1. Introduction

Cluster headache (CH), with a lifetime prevalence of 0.12% [1], carries a high personal burden to those affected. The pain is severe or very severe, unilateral, sharp and sometimes pulsating or pressing, and felt behind and/or around the eye or at the upper teeth, jaw, ear, or shoulder. It occurs in attacks lasting for 15 to 180 min, from once every other day to eight times per day [2]. About 80% of CH patients have the episodic form in which the attacks are restricted to susceptibility periods or “bouts” separated by remission intervals of at least three months. For ca. 20% of patients, however, the disorder is chronic, with the remission periods being shorter than three months or absent altogether [3].

The attacks are noteworthy for unilateral autonomic symptoms—lacrimation, rhinorrhea, miosis, ptosis, and forehead sweating—and by prominent agitation, including 50% of sufferers who report hitting a wall or hitting themselves in the head during an attack [4]. The agitation is usually separate from suicidal intention [5], but the pain is often excruciating—it is one of the most painful disorders known to humanity [6]—and 55–64% of sufferers do in fact report a history of suicidal ideation [4,7]. Also, 16–17% report having lost a full-time job due to CH [4,8]), a figure that would be higher were it not for the tendency of CHs to occur at night [4]). The urgent quest for better preventive and acute treatments is impeded, however, because much of the underlying pathophysiology remains unknown [9].

The modern understanding of CH focuses on the trigeminovascular system and the trigeminal autonomic reflex as effectors of the attack and a central permissive state centered on the hypothalamus [10]. The importance of the trigeminovascular system is supported by increases in calcitonin gene-related peptide (CGRP) levels in saliva and plasma during an attack [11,12,13], elicitation of an attack by infusion of CGRP [14], and by the effectiveness of subcutaneous sumatriptan and intranasal zolmitriptan for acute treatment [15]. Nonetheless, plasma CGRP levels, although higher within a cluster period than without, are lower than in healthy control subjects in both states of episodic CH and lower also in chronic CH [16]. Galcanezumab anti-CGRP treatment is effective for episodic but not chronic CH [17,18], and fremanezumab does not seem to be effective for either [19]. These suggest that trigeminovascular activation is not causal in the disorder but rather part of the downstream signaling cascade [16].

The importance of the trigeminal autonomic reflex is supported by the facial autonomic symptoms that are part of the diagnostic criteria [2] and by the efficacy of high-flow oxygen inhalation, and the possible efficacy of sphenopalatine ganglion stimulation, for terminating the attack [15,20].

Data supporting the hypothalamus include the circadian periodicity of the attacks and the circannual patterning of cluster bouts, neuroendocrine differences (e.g., lower melatonin levels in people with CH), and PET evidence of ipsilateral posterior hypothalamic activation during CH attacks [10,21]. Recent imaging studies point in particular to the enlargement of the anterior hypothalamus [22], an area that includes the suprachiasmatic nucleus (SCN) and the paraventricular nucleus (PVN), with stronger evidence in chronic CH for the PVN specifically [23]. Nonetheless, the involvement of the PVN in other forms of visceral pain such as angina and the pain in irritable bowel syndrome and pancreatic cancer [24,25,26] raises the possibility that the hypothalamus, too, is a downstream effector rather than initiator of the attack [26,27].

Moreover, the hypothalamic model so far has had limited therapeutic impact. With the exception of melatonin, none of the current preventive, bridging, or acute treatments now in clinical use is known to affect the hypothalamus [28]. Somatostatin analogs remain in early development—their site of action may or may not be the hypothalamus [10]—and deep brain stimulation of the hypothalamus, although logical, and effective in case reports, is a risky and invasive procedure deemed probably ineffective in American Headache Society guidelines [15]. Thus, despite its strong evidentiary basis, the role of the hypothalamus has so far been only weakly useful in translational medicine.

How can this translational gap be bridged? To address this, we will first ask a more fundamental question: What activates the hypothalamus to initiate an attack? To answer this latter question, we will turn to a theory first proposed by Lee Kudrow in 1983: that CHs are a dysfunctional response to hypoxia [29].

Our goal will be to explain the existing data on CH insofar as possible, identify gaps in current knowledge, and derive a specific program of basic and translational research to shed further light on this disorder.

## 2. Kudrow’s Carotid Body Theory

Kudrow proposed that during cluster periods, diminished sympathetic outflow and an overabundance of parasympathetic firing, thought to be due to hypothalamic dysfunction, caused the carotid bodies (CBs) to be less sensitive to oxygen desaturation. (The CBs, near the carotid artery bifurcations on each side of the neck, are chemosensory organs that detect the oxygen concentration and chemical composition of blood approaching the brain.) This impaired autoregulation, leading to prolonged hypoxemia, and caused CB hypersensitivity (analogous to denervation hypersensitivity), such that the prolonged hypoxemia triggered the CH as an overly zealous response to hypoxia [29,30].

Several streams of evidence seemed supportive. Although prevalence estimates vary widely [31,32], obstructive sleep apnea appears to be ca. eight times more common in CH than in the general population [33], and Kudrow found that 60% of CH episodes followed periods of sustained nocturnal desaturation, particularly during REM sleep when respiration may be suppressed [34]. Moreover, there are case reports of cluster-like headaches in carotid paraganglioma [35] and carotid dissections [36], both of which can affect the CB and, without the facial autonomic component, in polycythemia vera, a blood disorder that can arise from hypoxic signaling [37]. Similarly, CHs can be triggered by high altitude [38]. Diminished nocturnal lipolysis [39], too, may be an adaptation to hypoxia as fatty acids provide energy via oxidative (oxygen-dependent) phosphorylation.

Experimentally, sublingual nitroglycerin, a known CH trigger, causes sustained hypoxemia that does not spontaneously self-correct during a cluster period (but that does self-correct in CH patients in remission and in control subjects) but rather that culminates in a CH attack [40]. Of note, it is the duration rather than the degree of desaturation that seems to predict an attack [30].

Interest in the CB model ended after a series of small empirical trials in the 1990s, in which arterial oxygen saturation was measured before and during an attack [41] or in which CH patients inhaled air with reduced [42,43,44,45] or no [46] oxygen. The studies were consistent in showing higher baseline ventilation during a cluster period [41,42,43,46], implying tonic low-grade activation of the hypoxia system, and a tendency towards lower oxygen saturation at baseline [41]. This tonic activation tended to be additive with the activation in a low-oxygen environment [46] and protected against arterial oxygen desaturation [44,45], a protection seen naturalistically during a CH attack [41].

The studies were regarded as disconfirming the CB theory because they found neither greater CB responsiveness (the slope of the change in ventilation in relation to oxygen saturation was the same in CH patients as in control subjects [46]) nor consistent triggering of CH by hypoxia [42,43,45]. The CB theory was dismissed and now seems mostly forgotten. To the best of my knowledge, it is not mentioned in any recent review of CH pathophysiology or treatment. Hypoxia itself, however, has not been forgotten: Since the early 1990s, a great deal has been learned about hypoxic signaling because of its importance to numerous diseases including atherosclerosis, neurogenic hypertension, congestive heart failure, neovascular retinal diseases, and the adaptation of cancer cells to the tumor microenvironment. A great deal, too, has been learned about CH. Therefore, let us draw on this knowledge to update the hypoxia theory and connect it to the many aspects of CH.

In so doing, we will depart from Kudrow’s classic theory in three ways. First, we will remain agnostic about whether the heightened hypoxic signaling is due to an altered threshold or actual, perhaps transient and localized, hypoxia not well captured by systemic measures. We will see that most of the evidence favors the former hypothesis but that there are a few findings that seem best explained by the latter. Second, rather than remain with Kudrow’s biphasic model, in which the CBs are relatively insensitive to lower levels of hypoxia but supersensitive to levels above a certain threshold, we will adopt the simpler premise that hypoxia detection is elevated throughout. Third, we will dispense with the assumption that sensitivity to hypoxia is due to an autonomic imbalance initiated by the hypothalamus. By treating sensitivity to hypoxia as the primary problem, we will be able to derive new approaches to treatment and shed light on previously obscure features of CH.

First, however, let us examine how hypoxia is detected and communicated to the brain.

## 3. Hypoxia Signaling Cascade

### 3.1. Anatomy

**Figure 1:**
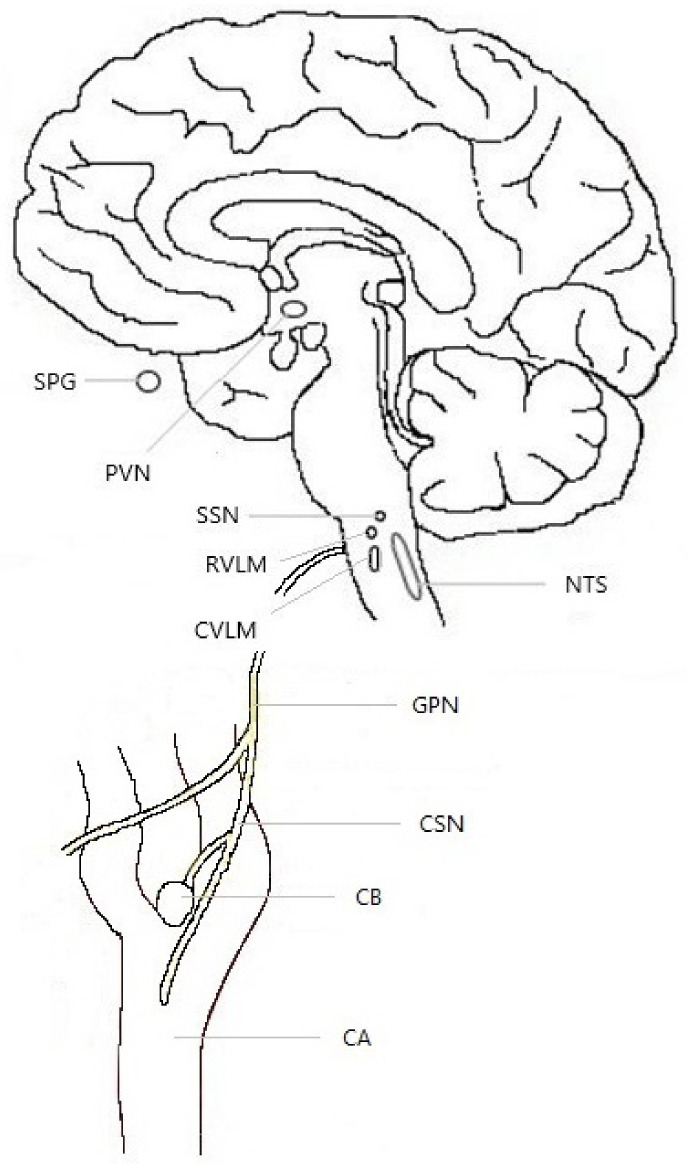
Structures involved in hypoxia detection and response, and in cluster headache. Abbreviations: CA—carotid artery; CB—carotid body; CSN—carotid sinus nerve; CVLM—caudal ventrolateral medulla; GPN—glossopharyngeal nerve; NTS—nucleus tractus solitarius; PVN—paraventricular nucleus of the hypothalamus; RVLM—rostral ventrolateral medulla; SPG—sphenopalatine ganglion; SSN—superior salivatory nucleus.

The CBs are comprised of clusters of chemosensory glomus cells and surrounding glial-like cells. The CB signals through the carotid sinus nerve to the brainstem respiratory and cardiorespiratory centers, particularly the caudal portion of nucleus of the tractus solitarius (NTS), and the rostral and caudal ventrolateral medulla (RVLM and CVLM). These three brainstem structures are interconnected and all three project to the (autonomic-regulatory) paraventricular nucleus of the hypothalamus (PVN) [47,48,49]. In turn, the PVN projects back to the NTS [50]. This feedback circuit maintains baseline respiration frequency, supports increased respiration in response to hypoxia [47,50], and presumably allows for feedback amplification (see Figure 1).

### 3.2. Mechanisms of Detection

In the CB, oxygen levels are detected primarily by four overlapping mechanisms. Understanding them will provide clues, discussed at the end of this paper, for novel ways to prevent and treat CH.

*NADH and Reactive Oxygen Species (ROS)*. The mitochondrial electron transport chain (ETC) depends on oxygen as the final acceptor at Complex IV. In an oxygen deficit, Complex IV fails. Electrons back up and, singly or in pairs, join directly with oxygen to form the reactive oxygen species (ROS) superoxide and hydrogen peroxide, respectively, particularly at Complex I. As Complex I converts NADH to NAD^+^, NADH levels rise as electron transport slows. NADH and ROS seem to be the key hypoxia signaling molecules within the CB [48].

*Hypoxia-Inducible Factor-1, -2, and -3 (HIF-1, HIF-2, HIF-3)*. The hypoxia-inducible factors are a family of isozymes having generally similar and overlapping functions but different tissue distributions [51]. They are heterodimers of HIF-1β, which is constitutively present, and HIF-1*α*, HIF-2 *α*, or HIF-3*α*, which are oxygen-labile. The -1*α*, -2*α*, and -3*α* subunits are constantly being produced and just as quickly removed by prolyl hydrolase domain (PHD) enzymes that mark them for degradation by the ubiquitin–proteosome system [51]. The PHD enzymes, however, depend on oxygen, iron, and alpha-ketoglutarate, and therefore under oxygen deprivation, HIF-1α, HIF-2α, and HIF-3α build up. Each of them binds to HIF-1β, and the resulting heterodimers translocate to the nucleus. There, with coactivator p300/CBP, the heterodimer binds to the hypoxia response element of the promoter region of certain genes, increasing their transcription. In glomus cells, HIF-2α is particularly abundant and upregulates genes encoding parts of Complex IV [48]. These components may reduce the ability of Complex IV to bind oxygen, making the ETC more sensitive to hypoxia [48].

*Succinate*. In hypoxia, cellular levels of succinate rise. In particular: (1) The buildup of NADH reduces the functioning of pyruvate dehydrogenase, so instead of being converted into acetyl-coenzyme A for entry into the tricarboxylic acid cycle, pyruvate is converted into oxaloacetate, which is ultimately converted into succinate [52]. (2) At low oxygen, the ETC slows, including at Complex II, succinate dehydrogenase (SDH), which metabolizes succinate. Thus, a buildup of succinate signals hypoxia. Oxidative stress compounds this as alpha-ketoglutarate is nonenzymatically oxidized to succinate [52].

**Figure 2:**
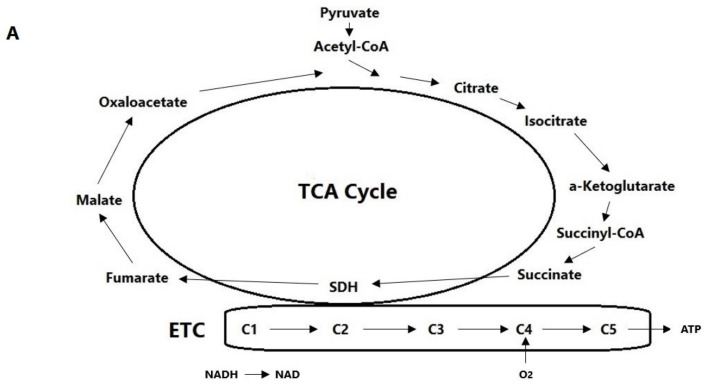

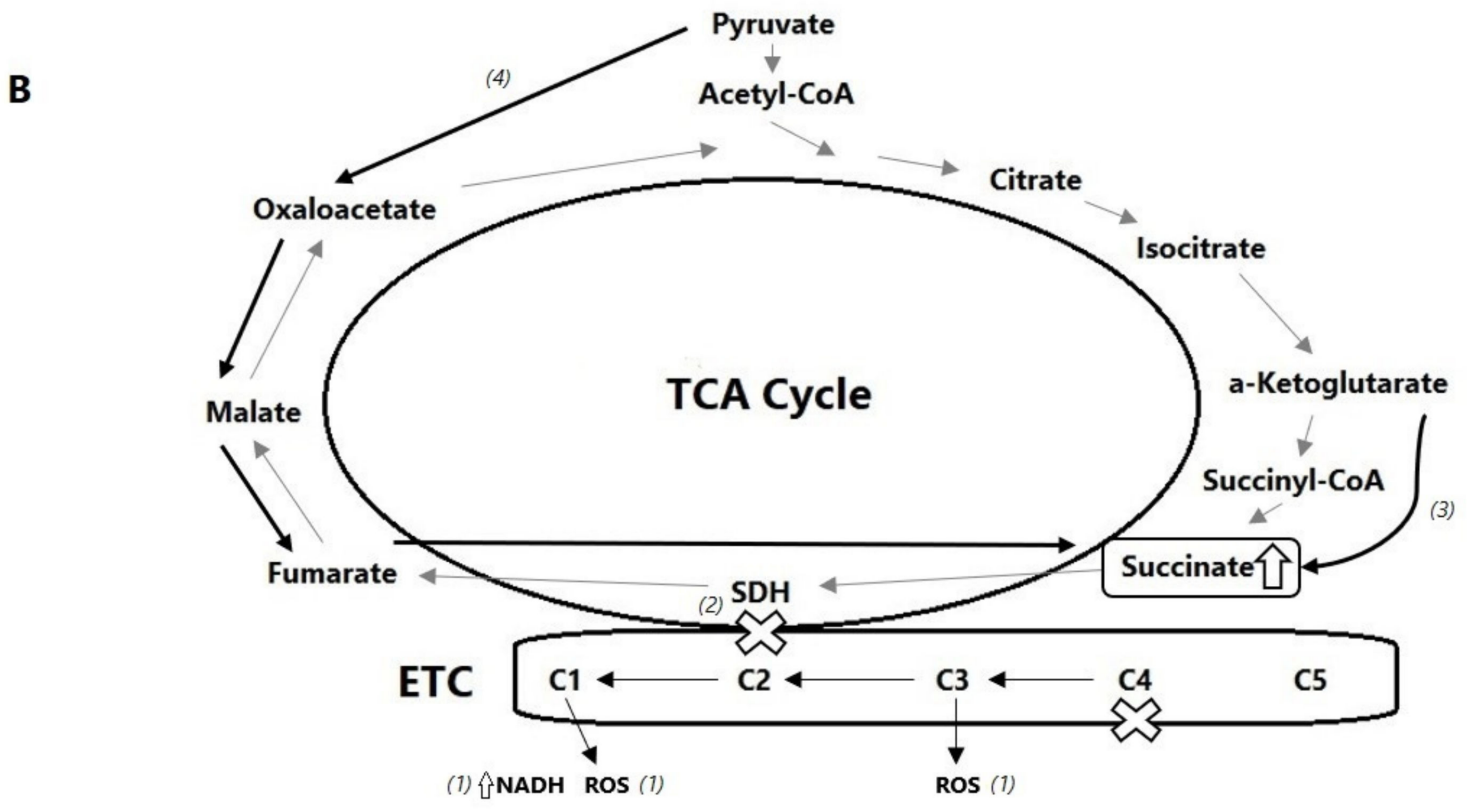
The tricarboxylic acid cycle and electron transport chain in normoxia (**A**) and hypoxia (**B**). In hypoxia (1), the electron transport chain functions in reverse, generating reactive oxygen species at Complex 1 and Complex 3, and allowing NADH to accumulate at Complex 1; (2) Complex 2/succinate dehydrogenase is impaired in the forward direction, causing succinate to accumulate; (3) α-ketoglutarate is nonenzymatically converted to succinate; and (4) pyruvate is preferentially routed through pyruvate carboxylase to produce oxaloacetate, causing some reverse flux through the latter part of the tricarboxylic acid cycle. As a result of (2)–(4), succinate builds up. Reactive oxygen species, NADH, and succinate signal the presence of hypoxia. Abbreviations: C1 to C5—Complex 1 to Complex 5; ETC—electron transport chain; NAD—nicotinamide adenine dinucleotide; NADH—nicotinamide adenine dinucleotide (reduced form); ROS—reactive oxygen species; SDH—succinate dehydrogenase; TCA—tricarboxylic acid cycle.

*Pyruvate Carboxylase*. Pyruvate carboxylase is abundantly expressed in the CB, where it may support hypoxia detection by maintaining NADH levels above a certain threshold [48] (see Figure 2).

### 3.3. Sensitization

When hypoxia is prolonged for hours or days, the CBs become sensitized, termed acclimatization, giving a stronger response to the same level of hypoxia. Underlying this anatomically is hyperplasia of the CB and structural changes in its chemosensory glomus cells [53].

Neurochemically, sensitization is a product of inflammation by way of endoplasmic reticulum (ER) stress. Important processes for proper protein folding in the ER, such as the formation of disulfide bonds between cysteine residues and the hydroxylation of proline residues, require oxygen. In hypoxic conditions, misfolded proteins accumulate, triggering ER stress [54]. Moreover, HIF-1α and -2α sensitize the ER stress response [54]. The increased ROS production in the mitochondria also plays a role, as oxidatively damaged mitochondrial proteins trigger the mitochondrial unfolded protein response, with additive effects [54]. In ER stress, the ER itself produces reactive oxygen species (ROS) [55]. The ER stress and ROS then initiate inflammation via the nuclear factor-κB (NF-κB) transcription factor and two branches of signaling by mitogen-activated protein kinases (MAPKs)—extracellular signal-regulated protein kinase 1/2 (ERK1/2) and c-Jun N-terminal kinase (JNK) [56].

Thus, at the cellular level, hypoxia, like other stresses, is an inflammatory stimulus. There is increased expression of toll-like receptor 4 (TLR4) and production of nitric oxide and the proinflammatory cytokines TNF-α, IL-1β, and IL-6 [57]. The inflammation promotes sustained hypoxic signaling as NF-κB increases the expression of HIF-1α [57]. Inflammation within the CB also seems to produce a chronic state of oxidative stress, in part through downregulation of antioxidant enzymes [58].

The prolonged hypoxic signaling itself causes sensitization. At the receiving synapses, hypoxic signaling that is sustained over days causes an increase in AMPA- and NMDA-type glutamate receptors [59]. A result of all this is tonic afferent drive to the NTS [60] and, like the CB, the downstream NTS–PVN–RVLM complex shows sensitization. Thus, in animal models of chronic intermittent hypoxia, the increased sympathetic drive to the respiratory centers is not reversed if the CBs are then removed surgically, suggesting that the downstream consequences of hypoxic signaling can be maintained independently of continued hypoxia [61]. Ultimately, chronic CB activation itself causes oxidative stress in the PVN [62].

Let us now examine each of the aspects of CH in light of hypoxia, starting with neuroimaging, genetics, and neurochemistry, and progressing to clinical features.

## 4. Cluster Headache and Hypoxia: Neuroanatomy, Neurochemistry, and GWAS Genetic Studies

### 4.1. Neuroanatomical Overlap

A role for the hypothalamic PVN in CH is supported by evidence that it has increased volume in the episodic and chronic forms of the disorder but not in migraine [22,23]. The relevance of signaling from the NTS to the PVN is supported by the efficacy of transcutaneous vagal nerve stimulation for CH [63], which is thought to work by altering this signaling [64].

Particularly in its corticotropin-releasing hormone neurons, the PVN receives direct and possibly bidirectional projections from the SCN pacemaker region [65] and in turn impresses a circadian periodicity on metabolism and hormonal secretion [66]. The PVN also projects to the ventrolateral periaqueductal grey [47], an area implicated in pain. Not surprisingly, then, the PVN seems to play a role in visceral pain [24,25] and projects to the trigeminal nucleus, influencing pain thresholds for the head [67,68]. Moreover, through projections to, successively, the superior salivatory nucleus [69], the sphenopalatine ganglion, and the facial nerve, the PVN may also account for the facial autonomic symptoms of CH [10,70]

However, as we have seen, the PVN, particularly the caudal part and the corticotropin-releasing hormone positive neurons of the medial parvocellular division, and the NTS, are also key structures in the hypoxia response network [71]. Not surprisingly, then, hypoxic signaling seems to directly facilitate pain transmission in the trigeminal ganglion [72]. Succinate and HIF-1α increase the expression and function of the nociceptive TRPM2 ion channel in the trigeminal ganglion of mice, raising periorbital pain sensitivity and susceptibility to nitroglycerin-induced migraine [72]. Hypoxia also activates the trigeminal nucleus caudalis (TNC) [73]. The mechanism is not known, but excitatory input to the TNC seems to be increased by chronic intermittent hypoxia as indicated by a greater number of noradrenergic terminals [74]. Also, there may be selective damage to the (sympathetic) locus coeruleus [75], which might have bearing on the parasympathetic dominance found in CH.

### 4.2. Cluster Headache Genetics

To understand hypoxic signaling in CH more thoroughly, it is helpful to examine the genes that have been implicated in CH in genome-wide association studies (GWAS). These studies have so far identified 8 loci in the vicinity of 16 genes. The strongest evidence in CH is for the 10 genes UFL1, CAPN2, PLCE1, DUSP10, SATB2, CFTR, MERTK, LRP1, FBLN7, and TMEM87B, with weaker evidence for another 6 genes: FHL5, NDUFAF4, SLC20A1, KLHL32, CAPZA2, and ST7 [76]. These genes appear to play a role in the cell’s response to hypoxia.

*FHL5* encodes a member of the *Four and a Half LIM* protein family, the LIM domain referring to zinc finger motifs whose cysteine residues support protein–protein interactions [77]. These interactions help regulate such aspects of cellular functioning as signaling and transcription [77]. In particular, FHL1, FHL2, and FHL3 appear to interfere with hypoxic signaling by binding to HIF-1α, preventing its dimerization with HIF-1β to form the HIF-1 transcription factor [77]. Although FHL5 does not seem to have been studied, it appears that a single full LIM domain is sufficient for these effects [77], implying that FHL5 also impedes hypoxic signaling.

*UFL1 (ubiquitin fold modifier ligase-1)*. Inflammation (NF-κB) increases the transcription of HIF-1α and contributes to hypoxic signaling [57]. UFL1 suppresses TLR4/NF-κB inflammatory signaling [78] and helps prevent ER stress from the buildup of misfolded proteins [78]. UFL1 also likely participates in a ubiquitin-like degradation of HIF-1α, parallel to the classical ubiquitin–proteosome system [79].

Thus, decreased functioning of either FHL5 or UFL1, or both, would be expected to cause a tonic increase in hypoxic signaling at normoxia. Because this effect is not oxygen-dependent, it would not change the slope of the oxygen saturation–neural response function but rather would be additive to the effects of hypoxia. This fits with the hypoxia studies of CH noted above.

*CAPN2* encodes *Calpain 2*, a calcium-dependent protease that contributes to cell signaling and regulation by cleaving other enzymes [80]. It is activated under hypoxia [81] and cleaves filamin A into a fragment that HIF-1 uses to navigate to the nucleus to initiate the hypoxic response [82]. In SH-SY5Y neurons in vitro, calpain-2 also causes ER stress and JNK1/2 inflammatory and apoptotic signaling in response to hypoxia [83]. Thus, CAPN2 likely facilitates both the hypoxic response and its sensitization.

*NDUFAF4,* encoding *NADH Dehydrogenase-Ubiquinone Complex 1 Assembly Factor 4*, participates in the assembly and function of Complex 1 of the ETC [84]. As Complex 1 consumes NADH and generates ROS, the main intracellular signaling molecules for hypoxia in the CB, alterations in Complex 1 activity affect the sensitivity of the hypoxia detection mechanism [48]. In human astrocytes, NDUFAF4 is among the most downregulated genes in response to hypoxia [84].

Other genes seem to encode links between hypoxia and inflammation.

*SLC20A1 (Solute Carrier 20A1),* at least in mouse chondrocytes, localizes primarily to the ER [85]. There it is downregulated under hypoxia, triggering the unfolded protein response [85] and ER stress, the first step in inflammatory signaling.

*PLCE1* encodes *Phospholipase C, Epsilon-1.* In rodent heart cells under hypoxia in vitro, and in rat hearts subjected to ischemia–reperfusion in vivo, PLCE1 mRNA and protein levels increase [86]. PLCE1 then phosphorylates P38 and ERK1/2, which in turn phosphorylate the P65 subunit of the NF-κB transcription factor [86]. This activates NF-κB-mediated inflammation [86].

*DUSP10*. *Dual Specificity Phosphatase 10* has an effect in hypoxia complementary to PLCE1. By removing phosphate groups from mitogen-activated protein kinases (MAPKs), it downregulates inflammation [87]. In mouse cortical neurons in vitro, DUSP10 is upregulated in response to 3 h of hypoxia and suppresses apoptosis by inhibiting p38/JNK stress signaling [88].

*SATB2 (Special AT-rich Sequence Binding Protein-2)*. SATB1 and SATB2 are transcription factors influencing the expression of numerous genes through the epigenetic reorganization of chromatin [89]. In particular, SATB1 promotes adaptation to hypoxia and dampens the neuroinflammatory response to hypoxia by restraining the JNK1 and NF-κB transcription factors [89]. At least in mice, SATB2 is expressed in numerous brain locations including the PVN [90].

*CFTR (Cystic Fibrosis Transmembrane Conductance Regulator)* is widely expressed and seems to have broad antioxidant, anti-inflammatory (on JNK, ERK, and NF-κB signaling), and anti-apoptotic effects [91,92]. Because hypoxia favors the production of ROS [93], and in turn, ROS amplify the detection of hypoxia [57], CFTR, if present in the hypoxia network, may be hypothesized to downregulate hypoxic signaling and to protect neurons under hypoxic conditions.

*MERTK (Myeloid-Epithelial-Reproductive Tyrosine Kinase)*. In microglia, MERTK is thought to play a role in neuroinflammation via ERK1/2 signaling and synaptic pruning [94], shifting the microglia towards the anti-inflammatory M2 polarization state [95]. The disruption of MERTK leads to sustained inflammation and increased neurological damage after experimental brain injury [95]. Conversely, HIF-1α, by upregulating glycolysis, cleaves MERTK to an inactive form [96]. Thus, MERTK seems well positioned to decrease inflammation under hypoxia, as has been demonstrated for its ligand, galectin-3 (discussed below).

*LRP1* (*Low-Density Lipoprotein Receptor-Related Protein 1*) reduces the inflammatory response of mouse astrocytes and microglia, including the production of TNF-α, IL-1β, and IL-6, by suppressing the p38, JNK, and NF-κB pathways [97]. This effect is barely seen under baseline conditions but becomes prominent in the presence of an inflammatory stimulus [97]. In the liver, LRP1 mRNA and protein levels appear to be increased by HIF-1 [98].

*FBLN7* (*Fibulin-7*) is a recently discovered cell adhesion molecule found primarily in the extracellular matrix [99]. It is anti-inflammatory in monocytes, macrophages [99], and neutrophils, the latter in an ERK1/2-dependent manner [100], and thus might have similar effects in microglia. It inhibits angiogenesis [99] and thus might inhibit the upstream angiogenic switch, HIF-1α. A related molecule, fibulin-5, is in fact induced by hypoxia [101]. Nonetheless, little is yet known about FBLN7. Similarly, little is known about the remaining genes, TMEM87B, KLHL32, ST7, and CAPZA2.

Taken together, it is tempting to speculate that the genetic disturbances underlying CH may cause lower thresholds for the detection of hypoxia, increased signaling when hypoxia is present, and sensitization of the pathways for hypoxia detection and response.

### 4.3. Neurochemistry: Neurotransmitters and Neuropeptides

*Galectin-3*, an endogenous ligand of MERTK, has serum levels eight-fold higher in CH patients during and outside of a cluster period than in control subjects, and galectin-3 is found in the rat trigeminal ganglion [94]. Of note, the promoter region of the galectin-3 gene contains two hypoxia-responsive elements, and the expression of the gene is increased by HIF-1 [102]. Within the cell, galectin-3 facilitates survival under hypoxic conditions by promoting antioxidant defense, stabilizing mitochondria, and by blocking apoptosis [102,103]. Outside the cell, in the extracellular matrix, galectin-3 also promotes angiogenesis and cell migration out of the hypoxic environment and, interestingly, increases apoptosis in this context [103]. Among the oxidants from which galectin-3 protects the cell is nitric oxide [104].

*Vasoactive Intestinal Peptide (VIP)*. VIP is a neurotransmitter in the parasympathetic sphenopalatine ganglion [105,106]. Serum levels rise during CH attacks and correlate with the extent of cranial autonomic symptoms [12]. Moreover, VIP infusion can induce cluster-like attacks during a cluster period and in chronic CH patients [106], perhaps by eliciting a rise in serum CGRP, as seen in migraine [107].

VIP may help counter hypoxia. In a rat model, the intracerebroventricular administration of VIP helps reverse ischemic brain damage following reperfusion by improving blood supply, preventing the apoptosis of microvascular endothelial cells, and by phosphorylating endothelial nitric oxide synthase (eNOS), thus protecting and dilating blood vessels and increasing angiogenesis [108]. Interestingly, VIP also seems to downregulate HIF signaling in in vitro models of neuroblastoma and diabetic macular edema [109,110]. Naturalistically, this might serve to terminate hypoxic signaling as blood supply is restored so that the newly available oxygen is utilized fully. Similarly, by increasing blood flow to the CBs, VIP seems to attenuate their activation [111], perhaps accounting for the self-limiting nature of the attack.

*Pituitary Adenylate Cyclase-Activating Polypeptide-38 (PACAP38)*. PACAP38 is released by both sensory and parasympathetic nerves. Its structure overlaps with VIP, and the peptides have two receptors in common [110]. As with VIP, PACAP38 levels rise during attacks in episodic CH [12], and its infusion during a cluster period or in chronic CH elicits a cluster-like attack [106]. Outside of a cluster period, however, PACAP38 levels are lower than in control subjects [112].

PACAP38 stimulates glomus cells in the CB and, in animal models, is required for normal ventilation [111]. PACAP38 and its receptor, PAC1, are upregulated in the CB in response to hypoxia [113]. In the brain, PACAP38 promotes nitric oxide production, vasodilation, and angiogenesis, and has shown promise for neuroprotection in animal models of transient or permanent ischemia [114], implying a protective role in hypoxia.

*Calcitonin Gene-Related Peptide (CGRP)*. CGRP is a strongly vasodilatory molecule released by sensory nerves. As with VIP and PACAP38, CGRP levels rise during CH attacks and infusion during a cluster period or in chronic CH can elicit an attack [14]. As noted, CGRP levels are lower in chronic CH and in episodic CH within and, especially, outside of a cluster period [16]. Through nitric oxide production, vasodilation, and trophic signaling at the endothelium, CGRP protects against ischemia, reducing lesion size and behavioral deficit [114]. Here, too, a protective role in hypoxia seems likely.

*Trace Amine: Tryptamine*. Chronic CH, measured 4+ hours after an attack, is characterized by high serum levels of the trace amine and tryptophan derivative tryptamine, as well as elevated levels of epinephrine, norepinephrine, and the tyrosine-derived trace amine tyramine [115]. Of note, the enzyme that produces tryptamine, tryptophan decarboxylase, is the only major tryptophan-metabolizing enzyme that does not require oxygen [116]. Not surprisingly, flux through tryptophan decarboxylase increases under hypoxic conditions, leading to increased tryptamine levels in the serum, brain, and liver of mice [116].

*Trace Amine: Tyramine*. Tyrosine metabolism is also perturbed in CH. Plasma tyramine levels are elevated in episodic CH, even in remission periods, are higher still during active periods, and are increased further in chronic CH [117]. Note that tyrosine hydroxylase, catalyzing the first, rate-limiting step in producing dopamine, norepinephrine, and epinephrine, requires oxygen to function while the alternative pathway through tyrosine decarboxylase, producing tyramine, does not. Thus, hypoxic conditions favor the production of tyramine over norepinephrine. The situation is complex, however, as long-term hypoxia causes an adaptive increase or decrease, depending on the brain region, in the expression of tyrosine hydroxylase [118,119].

*Nitric Oxide*. Nitric oxide synthesis appears to be upregulated in CH patients, more so during a cluster period [115,120,121]. This may account for the pain and vasodilation of a CH [121], consistent with the triggering of CH by nitroglycerin [5].

The combination of prolonged hypoxia and IL-1β, which may be elevated in CH [122,123], increases the expression of inducible nitric oxide synthase (iNOS) in several cell types, including macrophages [124]. iNOS is able to produce ca. 1000 times more nitric oxide than the endothelial and neuronal constitutive forms of the enzyme [124], making it a potential source for the elevations seen in CH. Moreover, during hypoxia, the brain imports nitrite, which can then be converted to nitric oxide by deoxyhemoglobin [125].

The neurochemical changes in CH are intriguing in that they imply not only exaggerated hypoxic signaling but actual hypoxia responsible for the elevated signaling. The location and timing of such hypoxia, however, remains unknown.

In addition to the data on neurochemistry, neuroanatomy, and genetics, the hypoxia theory provides a framework for understanding the distinctive clinical features and current pharmacology of CH. Let us now review these.

## 5. Cluster Headache and Hypoxia: Clinical Features

### 5.1. Behavioral Manifestations

Behaviorally, the most striking feature of a CH attack is intense agitation and restlessness—a compulsion to move—manifesting for example as walking or rocking, banging one’s head or hands, and sometimes as aggression [126], distinguishing it from migraine, other primary headaches, and many other pain conditions.

Montagna et al. [127] suggested that while migraine is experienced as a type of visceral pain, arising from within the body and eliciting motor quiescence in the service of rest and recuperation, CH is experienced as exteroceptive pain—an attack from outside—eliciting the behavioral responses of fight or flight. Alternatively, Manzoni et al. felt that the positions of CH patients seemed as if they were looking for a change in posture to relieve the pain [128]. To Graham, the behavior during attacks seemed frantic [129]. In Torelli and Manzoni, 48% of CH patients described themselves as trying “incessantly to find environmental conditions that could relieve their pain” ([126], p. 116). This is reminiscent of the behavioral response to hypoxia, which includes intense efforts to escape to an environment in which oxygen is more plentiful [61]. Indeed, coordination of the response to hypoxia is one of the oldest and most conserved functions of the nervous system, identifiable in roundworms and fish [61]. Thus, similar to inhaling elevated concentrations of CO_2_, hypoxia elicits vigorous escape behaviors in rats [130] and panic-like symptoms in susceptible people [131]. Of course, fight-or-flight and escape from hypoxia are not mutually exclusive. In the animal kingdom, being attacked and suffocated is likely one of the more common causes of hypoxia.

### 5.2. Relationship to Cigarette Smoking

A strong risk factor for CH is exposure to tobacco smoke [4]. In the United States, 83% of CH sufferers have a personal history of cigarette smoking, of whom 85% were also exposed to high levels of tobacco smoke in childhood [132]. Rozen has suggested that both forms of exposure may be important, with the early life exposure sensitizing the individual to the later effects of smoking [132]. Moreover, people with CH tend to smoke a higher number of cigarettes than control subjects who smoke, and the attacks in smokers are on average more numerous, painful, and longer-lasting [133].

A causative agent in cigarettes has yet to be found but candidates are nicotine, the heavy metal cadmium, and the toxin acrolein [132]. Cadmium, present at 1 mcg/cigarette, is readily absorbed into the body, where it has a half-life of 15–20 years. Animal models suggest that it could account for the low serum testosterone seen in CH, cause low cerebrospinal fluid levels of orexin (an antinociceptive hypothalamic peptide), and alter the circadian clock, which can increase CGRP production and release [132]. Acrolein, a reactive aldehyde, is present at 25–140 mcg/cigarette [134]. It is an oxidizing irritant that induces meningeal CGRP release via the TRPA1 ion channel [132].

Through several mechanisms, tobacco smoke facilitates sensitivity to hypoxia: (1) In humans, nicotine acutely sensitizes the CB to hypoxia [135], an effect confirmed with chronic cigarette smoke exposure in rats [136]. Thus, smoking and second-hand smoke exposure bias the system towards the over-detection of hypoxia. (2) Chronic tobacco smoke exposure in rats desensitizes the arterial baroreceptors [136], impairing compensation for changes in blood pressure. Because baroreceptors inhibit the CB, smoking would disinhibit the detection of hypoxia. (3) Acrolein inhibits several dehydrogenase enzymes, particularly SDH/Complex II. Some inhibition of Complex II is seen at 100 μM acrolein and 80% inhibition at 1 mM [137]. The resulting buildup of succinate can be a pseudo-hypoxic signal [138]. (4) Smoking increases ROS, key signaling molecules for hypoxia.

### 5.3. Triggers—Alcohol and Nitroglycerin

Alcohol is an acute trigger of CH [4]. It can create hypoxic conditions intracellularly and induces HIF-1α, particularly in the liver, because cytochrome P450-2E1 (CYP2E1), one of the enzymes that metabolizes alcohol, consumes oxygen [139]. Nitroglycerin, also an acute trigger of CH [11], via the production of nitric oxide promotes the nuclear accumulation of the HIFs by inhibiting PHD enzyme function [140].

### 5.4. Circadian Patterning of Cluster Headache Attacks

CH attacks typically occur at night, particularly between 1:00 and 3:00 AM [4]. This circadian patterning is generally taken as prima facia evidence for the role of the SCN in generating attacks. However, circadian variation is also an intrinsic feature of hypoxia detection. (Conversely, certain clock genes are induced by hypoxia [141].) In awake humans, chemosensitivity to CO_2_ and presumably O_2_ is low overnight and rises in the morning, peaking around 2:00 PM (assuming a midnight to 8:00 AM sleep schedule) [142]. The erythrocyte count also falls at night in people [143]. As a result of both of these, arterial oxygen saturation is lower at night. Cristancho et al. found that this nocturnal effect was stronger in males and, interestingly, peaked between 1:00 and 3:00 AM [144].

Intermittent hypoxia can also disrupt the entrainment of peripheral clocks in the chemoreceptive network (most likely the CB and NTS [145]) to the master clock in the SCN [146]. This could create a mismatch between sensitivity to hypoxia and the times when hypoxia is more likely to occur, causing at times an over-read. Thus, in mice, which are nocturnal, when the clocks are disrupted via knockout of the BMAL1 clock gene specifically in the chemoreceptive network, the ventilatory response to hypoxia decreases at night, and the daytime response (equivalent to nighttime in people) increases [145]. Thus, the circadian features of CH seem compatible with both actual hypoxia and with altered hypoxic thresholds.

### 5.5. Circannual Patterning of Cluster Bouts

Cluster bouts seem to be more common at certain times of the year, although there is quite a bit of divergence about when these times are [4,30,128,147,148,149] and there may be individual differences among people in circannual patterning. Despite its complexity, this patterning may help shed light on what activates a cluster period.

Seasonality implies an effect of the hypothalamic SCN but there are also points of intersection with hypoxia. Thus, although the percentage of oxygen in the air remains constant at ca. 21%, the amount of oxygen (e.g., as grams per cubic meter) varies with atmospheric pressure [150]. Except in equatorial regions, the partial pressure of atmospheric oxygen (pO_2_) varies with the season, averaging 10–15% lower in July and August than in January and February [150]. This might suggest greater risk of CH bouts in the summer. However, day-to-day variability in pO_2_ is greatest in the winter [150], which might tax the chemosensory system.

Further complexity arises from thermoregulation. Increases in core body temperature induce both an increase in CB output and increased responsiveness of the CB to hypoxia [151]. The resulting increase in ventilation may play a minor role in dissipating heat and maintain sensitivity to hypoxia despite decreased CO_2_ from slowed metabolism [151,152]. Hypothermia also increases CB firing, likely through increased blood CO_2_ from shivering and also through increased sympathetic activity [151]. Cold does not directly increase CB sensitivity to hypoxia, however, so the effects of heating are greater than those of cooling.

Thus, there are mechanisms by which the over-detection of hypoxia could be greater in some seasons than others, but whether this explains the circannual patterning of cluster bouts remains to be determined.

### 5.6. Autonomic Features

Prominent in CH are parasympathetic symptoms at the face, ipsilateral to the pain—lacrimation, rhinorrhea, forehead sweating, miosis, and ptosis. This cephalic trigemino-autonomic reflex is not well understood but a connection to hypoxia is implied by the effectiveness of high-flow oxygen in terminating it [153]. Hyperoxemia is an efficient means of silencing HIF signaling [154].

Systemically, there is evidence that CH attacks are more likely during times of parasympathetic dominance [155]. Moreover, contrary to the usual response to pain [156], heart rate slows during CH attacks [155]. This is all the more remarkable given decreased baroreceptor sensitivity in CH [157], which would make an increase in blood pressure from acute pain less effective at slowing heart rate.

As first noted by Kunkle and Anderson in 1960 [158], this heart rate response is reminiscent of the relative bradycardia elicited by cooling the face (diving reflex), compressing the eye (oculocardiac reflex), stimulating the nasal mucosa (nasopharyngeal reflex), or irritating the trigeminal nerve (trigeminocardiac reflex) [159]. All of these various trigemino-autonomic reflexes are thought to be adaptive mechanisms of oxygen conservation [160,161]. In particular, bradycardia may prevent cardiac damage under low-oxygen conditions [61].

### 5.7. Male–Female Ratio

CH has a male:female preponderance of ca. 2:1 currently [5] and 4.3:1 historically [1]. This decline in sex ratio may reflect increased smoking by women and/or past diagnostic confusion with migraine. The reason for male predominance of this disorder is not known. Interestingly, however, the administration of progesterone may improve arterial O_2_ and CO_2_ levels in COPD, sleep-disordered breathing, and chronic mountain sickness, and reduce the occurrence and severity of sleep apnea [162]. Sex [162] and estradiol [163] seem to alter HIF-1α levels, the direction depending on the specific tissue and model. Thus, the male preponderance of CH fits with a possible sexual dimorphism in hypoxia susceptibility and signaling.

### 5.8. Dietary Changes and Food Cravings

Nearly half of CH patients report a decrease in appetite during cluster periods and about 18% report cravings for sweets then [134]. The CBs are sensitive to hypoglycemia and participate in eliciting the compensatory increase in glucagon, cortisol, and adrenalin [164]. Moreover, there seems to be crosstalk between the oxygen- and glucose-sensing functions such that hypoglycemia is over-read in chronic intermittent hypoxia [164].

### 5.9. Comorbid Hypertension

People with CH appear more likely to have hypertension even after correcting for smoking, excessive alcohol intake, and higher body mass index [133]. Elevations in blood pressure are among the cardiopulmonary compensations for hypoxia set in motion by the CB, and excessive CB tone is a source of neurogenic hypertension [55].

## 6. Cluster Headache and Hypoxia: Current Pharmacology

Nearly all current CH treatments have been discovered empirically, with no certain connection to the pathophysiology of the disorder. They do, however, have interesting impacts on hypoxic signaling.

### 6.1. Preventive Treatments

*Verapamil*. The calcium channel blocker verapamil is a traditional mainstay for CH prevention [165], with a great deal of supportive clinical experience [166]. As with many other drugs of its generation, the quality of trials is low, garnering it only a Level C recommendation in the AHS guidelines [15]. The use of verapamil was based on the now-superseded vascular theory and the reasons for its efficacy are unknown. However, in vitro it suppresses the circadian rhythm, and in rats the related compound nimodipine reduces CGRP expression in the trigeminal nucleus caudalis [166]. In addition, however, in cardiac myoblasts in vitro, verapamil 20 μM (within therapeutic dose range) prevents the hypoxia-induced increase in HIF-1α levels and verapamil ≥10 μM lowers HIF-1α levels in normoxia [167].

*Melatonin*. Twenty-four-hour melatonin levels are lower in people with CH [168] and lower still during cluster bouts [169]. Melatonin secretion is suppressed by the mild hypoxia of long-distance air travel [170]. There is some evidence (Level C) that melatonin 10 mg in the evening reduces episodic CH [15,171].

Chen et al. found that physiologic levels of melatonin enhanced CB sensitivity and the ventilatory response of conscious rats to hypoxia [172]. Pharmacological levels, however, reduce HIF-1α protein levels, the transactivation of HIF-1 at the hypoxia response element, and the transcription of vascular endothelial growth factor (VEGF) as an exemplary HIF-induced gene [173]. In most but not all studies, the pharmacological dose was 10^2^- to 10^8^-fold greater than in Chen et al. At this higher level, HIF-1α may be destabilized by the antioxidant effects of melatonin [173] or, in theory, there could be a compensatory downregulation of melatonin receptors in the CB.

*Lithium*. Lithium is also a traditional mainstay preventive although, as with verapamil, it has only a Level C recommendation [15] because of the relative paucity of well-controlled trials. Its mechanism of action in CH is unknown, but from its efficacy in bipolar disorder, the presumptive attribution has been to its influence on neurotransmission (especially serotonergic) and on the circadian clock [174]. Compared to verapamil, its therapeutic effects arise more slowly.

In contrast to the other preventives discussed here, lithium has the potential to increase rather than decrease hypoxic signaling. At therapeutic levels, lithium inhibits glycogen synthase kinase-3β, an enzyme that participates in the degradation of HIF-1α [175]. Also, lithium ions form a complex with magnesium and ATP, prolonging purinergic signaling through the P2X receptor [176]. As the CB communicates the presence of hypoxia by releasing ATP and activating P2X receptors on the glossopharyngeal nerve [48], lithium would likely intensify the signaling. Moreover, by inhibiting ectonucleotidase, lithium increases adenosine levels [177], a molecule that facilitates acclimatization (sensitization) to chronic sustained hypoxia [178]. In theory, lithium might prevent CH through a longer-term desensitization of hypoxic signaling. Consistent with this, lithium seems to take at least 2 weeks to have a therapeutic effect [179]. Alternatively, lithium might produce an increase in ventilation that is protective against actual hypoxia.

*Histamine Desensitization*. In 1939, when Horton and coworkers proposed the diagnostic entity later known as CH, they reported that sustained remission could be robustly obtained with twice daily subcutaneous histamine at a dose apparently too low to trigger an attack [180]. Histamine desensitization has seen no double-blind trials, however, and is not mentioned in current treatment guidelines (e.g., [15,19]) or even as a last resort [181]. Clinically, however, there have been patient series with good results (e.g., [182]). Of note, mice deficient in the H1 histamine receptor show an impaired response to hypoxia [183]. Thus, the downregulation of this receptor through desensitization might indeed have a beneficial effect.

### 6.2. Acute Treatments

*Oxygen*. High-flow oxygen was originally suggested as an acute treatment for CH by Horton in 1952 [184], possibly for its vasoconstrictive properties [185], and it remains a mainstay, with Level A evidence [15]. It does not seem to reduce trigeminal pain signaling directly but rather indirectly by suppressing the parasympathetic component of the trigeminal autonomic reflex [153]. Not surprisingly, the hyperoxemia induced by oxygen effectively reduces HIF levels and interrupts hypoxic signaling [154].

*Prednisone*. Prednisone is used clinically for the acute treatment of CH, although the near absence of controlled trials makes its efficacy difficult to assess formally [15]. A related compound, dexamethasone, inhibits hypoxic signaling via the glucocorticoid receptor by blocking the translocation of HIF-1 to the nucleus [186].

*Intranasal Lidocaine*. This treatment, with level C evidence [15], blocks the parasympathetic sphenopalatine ganglion and likely interrupts the trigeminal autonomic reflex and trigeminovascular activation [187]. Thus, intranasal lidocaine likely works on a key effector mechanism of CH, downstream of hypoxic signaling.

*Triptans and Anti-CGRP Medications*. Subcutaneous sumatriptan and intranasal zolmitriptan each have level A recommendations in the 2016 AHS guidelines [15]. The anti-CGRP medication galcanezumab is supported for episodic but not chronic CH while, interestingly, fremanezumab seems efficacious for neither [19]. Triptans and galcanezumab inhibit trigeminovascular activation, an effector for the pain of CH, rather than targeting the upstream hypoxic signaling. In fact, they may work despite their upstream influence. At least in hyperglycemia, CGRP inhibits HIF-1 signaling in microvascular endothelial cells [188]. Similarly, sumatriptan succinate presumably helps despite the likelihood of a slight increment in hypoxic signaling from the succinate moiety.

## 7. Limitations and Directions for Future Basic Research

The hypoxia theory provides a unified account of numerous aspects of CH, but it also has important limitations that constitute directions for further research.

In this paper, episodic and chronic CH have been treated as two almost interchangeable manifestations of the same disorder but there may be important differences between them. For example, high-flow oxygen generally benefits a higher proportion of episodic CH patients than chronic [189], particularly chronic CH patients above 50 years old [190]. One possibility is that hypoxic signaling is more entrenched in the downstream NTS–PVN–RVLM complex, making it insensitive to a decrease in afferent input from the CBs. It might be possible to study this by combining pharmacological probes with vagal nerve stimulation, which is thought to specifically target NTS–PVN signaling. Alternatively, it may be the trigeminovascular response that becomes sensitized, as suggested by the numerically weaker response of chronic CH to intranasal zolmitriptan [191].

The processes that seem to be involved in CH are not unique to this disorder. Hypoxic signaling, ER stress, inflammation, and ROS likely play a role in other types of chronic pain [192]. CH appears to be distinguished by the extent of its dependence on hypoxic signaling, but studies contrasting its physiology with other types of pain will be enlightening. Similarly, it will be important to tease apart the sensitization of the trigeminovascular system, due to repeated attacks, from the tonic activation of the hypoxia system.

The neuroanatomical basis of CH in the hypoxia system requires verification. Data so far indicate that the PVN, a key component of the hypoxia network, controlling ventilation, has increased volume in chronic and episodic CH but not in migraine, projects to the TNC, and influences pain thresholds for the head. Similarly, hypoxia seems to facilitate pain transmission through the trigeminal ganglion and the TNC. However, these correspondences do not prove that hypoxia is driving the CH attack. More direct evidence might be obtainable by combining functional neuroimaging of the CH attack with the pharmacological facilitation and inhibition of hypoxic signaling.

A key challenge in CH, as in migraine, is to account for the unilateral pain and autonomic symptoms with a systemic theory. Experimentally, hypoxia seems to cause a lateralized decrease in frontal and temporal blood flow [193]. The lateralization seems to depend on time of day, season, and sex, with considerable interindividual variation even after these are accounted for [194]. However, to my knowledge there have not yet been studies of hypoxia and lateralized hypothalamic activation.

An ambiguity inherent in the current theory is whether CH is purely a disorder of exaggerated hypoxic signaling, as suggested by the GWAS results, or involves actual, perhaps transient and localized, hypoxia, as suggested by the circadian patterning and trace amine levels. One way of reconciling these may be to study the microbiome, which appears to play a role in migraine [195] but has not yet been studied in CH. The microbiota changes with hypoxia [196] and time of day [197], can produce large amounts of trace amines [198], and may facilitate hypoxic signaling by raising blood levels of acetate [199], an agonist of the olfr78 receptor in the CBs [200]. However, all of this would need to be examined empirically in CH.

A particular note of caution is the risk of confirmatory bias. This is seen especially in the analysis of the genes connected to CH. Because the effects of a single gene can be complex and pleiotropic, and can vary with cell type and experimental model, understanding it through the lens of a particular theory is inherently selective and therefore potentially biased. As a result, the hypoxia theory, despite its considerable explanatory power, rests largely on circumstantial evidence. In part due to the lack of an animal model for CH, there are no molecular demonstrations of heightened hypoxic signaling preceding or during CH attacks. This places greater emphasis on future clinical studies, and in particular on translational research that leverages the hypoxia theory for novel treatment directions.

## 8. Translational Research

The hypoxia model allows us to hypothesize several potential new treatments as appropriate research directions.

### 8.1. Pharmacological Treatments

Decreasing hypoxic signaling pharmacologically is a logical step. This could be accomplished in several ways:

*Inhibiting HIF-2*. HIF-2 adjusts the sensitivity of the CB rather than being essential for it to function at all. In December 2023 the FDA approved the first HIF-2α antagonist, belzutifan, a molecule that disrupts the binding of HIF-2α to HIF-1β, decreasing HIF-2 transcriptional activity. Belzutifan was approved for a type of renal cell carcinoma due to von Hippel–Lindau disease [201] but exploring its effects on CH may be enlightening. In clinical trials for cancer, a closely related compound, PT2385, eliminated CB responsiveness to hypoxia without a definite effect on baseline ventilation [53]. Nonetheless, the long-term effects on ventilation should be studied more (see Figures 1B and S3B in [53]), particularly given the risk of complications in sleep apnea [202], COPD [53], and respiratory depression from opiates [203]. Also important to study would be the risk of anemia [53,204] and the long-term impact on tissues such as intervertebral disks and stem cell niches, which are naturally hypoxic. However, the dose of HIF-2α inhibitors for CH will likely be much lower than that used for renal cancer.

*Antagonizing CB Signaling*. The glomus cells activate the glossopharyngeal nerve primarily by releasing ATP, and secondarily acetylcholine [48]. The ATP is an agonist at postsynaptic P2X receptors, suggesting that P2X3 antagonists currently in clinical trials (e.g., gefapixant for chronic cough) might have an effect in CH. However, the pharmacology of the chemoregulatory system is complex. Within the CB, purinergic (ATP) and angiotensin receptors seem to facilitate the cardiovascular responses, acetylcholine seems to facilitate and dopamine to inhibit the ventilatory response, dopamine may also inhibit the glycemic response, and there may be additional, yet-undiscovered specificity [205]. This suggests that anticholinergic agents and dopamine agonists might have benefit during cluster periods.

*ETC Complex I*. The genetic deletion of Complex I is embryonically lethal, but selective deletion in the CB in adulthood causes a loss of sensitivity to hypoxia [48]. This might suggest that Complex I inhibitors such as metformin could have a benefit in CH. However, the inhibition of Complex I, by causing a buildup of NADH, would also replicate some aspects of hypoxic signaling, so the effects would need to be empirically determined.

### 8.2. Nutraceutical Treatments

Several dietary constituents have defined effects on hypoxic signaling, but are so far mostly unexplored in CH.

*Thiamine*. Costantini et al. [206] describe sustained remission in a patient with chronic CH with vitamin B1 titrated to 500–750 mg/day, ca. 600 times the RDA. The authors hypothesize a local neural thiamine deficiency caused by abnormal transport or enzyme structure. The headaches recurred at a dose of 1000 mg/day. Very high doses of B1 can likely mimic a deficiency by causing a compensatory downregulation of thiamine-dependent enzymes [207]. In a fish model, moderate thiamine supplementation lowered the expression of HIF-1α and lowered the hypoxia-induced elevations in ROS and ER stress [208]. This is logical because both oxygen and thiamine are required for the functioning of the tricarboxylic acid cycle, such that their deficiencies require similar metabolic adjustments [209]. Mechanistically, the dysfunction of the thiamine-dependent pyruvate dehydrogenase complex causes intracellular pyruvate levels to rise. The excess pyruvate can then stabilize HIF-1α by competing with alpha-ketoglutarate, a required cofactor for the PHD enzymes [210]. The dysfunction of pyruvate dehydrogenase also causes pyruvate to be preferentially metabolized through pyruvate carboxylase, ultimately yielding succinate, a hypoxia-signaling molecule [52]. High-dose thiamine may compensate for these effects. However, there have been no studies of thiamine in CH beyond the single case report.

*Kaempferol*. Flavonoids affect all phases of HIF-1 signal induction including HIF-1α transcription, mRNA stability, protein stability and degradation, and binding to the hypoxia response elements of genes [211]. Particularly important, however, is that a wide range of flavonoids reduce HIF-1 activity by preventing its nuclear translocation [212,213]. Interest has focused especially on kaempferol, found in spinach, kale, and broccoli. This is because other flavonoids such as quercetin contain adjacent hydroxyl groups, allowing them to chelate Fe^3+^. Because iron is required for the degradation of HIF-1α, flavonoids such as quercetin and luteolin increase the amount of HIF-1α even while sequestering it in the cytoplasm [213]. Kaempferol, whose hydroxyl groups are not adjacent, lacks this quality [213]. Indeed, the anti-HIF-1α effect of kaempferol, at concentrations close to those achievable with diet, is sufficiently strong to be cytotoxic to liver cancer cells grown in hypoxic conditions [212].

*Other Polyphenols.* Curcumin, a component of the spice turmeric, may downregulate the activity of HIF-2α by decreasing the expression of HIF-1β [214], thus preventing the formation of the active heterodimer. This would also reduce HIF-1α signaling as it, too, requires dimerization with HIF-1β. A high dose of green tea polyphenols, after 6 months, although not 3 months, alters the rat gut microbiome to reduce succinate production by 60% [215].

*Beet Juice*. In one study, increasing serum levels of nitrate and nitrite by supplementing with beet juice reduced CB sensitivity to hypoxia in patients with obstructive sleep apnea [216].

*Riboflavin*. Succinate contributes to hypoxic signaling, suggesting that enhancing the breakdown of succinate through increased SDH activity might help. The cofactor of SDH is riboflavin (vitamin B2). Riboflavin supplementation has been used successfully to stabilize or improve the clinical symptoms of Complex II deficiency [217] and in preventing migraine but it does not seem to have been tried in CH.

*Antioxidants*. ROS seem to be the key signal for hypoxia within the glomus cells of the CB. However, oxidative stress also has a regulatory feedback function, causing the degradation of HIF-2α [58]. In a rat model, the sensitizing effect of chronic intermittent hypoxia on CB function was eliminated by antioxidants, including the flavonoid apocynin [218], the combination of losartan and allopurinol [218], and vitamin C [57]. There is no indication, however, that antioxidants can reduce sensitization once it has been established [57]. Thus, started early, antioxidants might have a preventive role for individuals at genetic risk for developing CH.

*Carnosine*. In vitro, carnosine has been shown to decrease HIF-1α protein levels in cardiac myoblasts (1 μM, well within the physiological range) [167], human colon cancer cells (50 and 100 mM) [219], human liver cancer cells (100 μM of a membrane-permeable derivative) [220], and in vivo in retinal ischemia in mice (1000 mg/kg, single treatment) [221]. The translation, however, may be complex. Through antioxidant activity, carnosine can likely preserve alpha-ketoglutarate, an obligate cofactor for HIF-1α degradation, thus lowering HIF-1α levels. However, carnosine can also raise HIF-1α protein levels by chelating Fe^2+^, also a required cofactor for HIF-1α degradation [222]. Which effect predominates seems to depend on cell type and experimental design.

### 8.3. Behavioral Treatments

*Breath Training*. A logical approach to CH is breath training (Susan McCrea, PsyD, personal communication). Respiration paced at six breaths per minute produces, through increased tidal volume, a reflexive decrease in chemoreflex sensitivity [223] that becomes more sustained with long-term practice [224]. Slow-paced breathing increases baroreflex sensitivity [223], which may contribute to the reduced hypoxic tone.

*Physical Exercise*. Activation of the baroreceptors diminishes the sensitivity of the CB to hypoxia [225]. Speculatively, this raises the possibility that physical exercise during a cluster period would ameliorate the attacks.

*Lifestyle*. There is no evidence so far that smoking cessation alters the course of CH [226]) but avoidance of smoking is wise in people genetically susceptible to CH. Also, the CB is chronically activated by hypercaloric diets, contributing to metabolic syndrome [227], potentially making diet and exercise relevant. Weight loss can help with sleep apnea as well, which might reduce triggering from overnight oxygen desaturations.

Of course, the possibility of new treatments based on hypoxic signaling should not be cause for overlooking the extant treatments based on the trigeminovascular system. For example, Haane and Koehler [228] report an excellent prophylactic response, including an end to the cluster period in some patients, from time-contingent transcutaneous stimulation of the supraorbital nerve.

## 9. Conclusions

The hypoxia theory of CH, proposed by Lee Kudrow in 1983 but neglected for the past 30 years, seems intriguing in the light of recent findings on CH and on hypoxia detection and sensitization. It sheds light on numerous features of CH that have been obscure until now, suggests a program of basic and translational research, and opens the possibility of new pharmacological, nutraceutical, and behavioral therapies for prevention and treatment of this disorder.

## Data Availability

No new data were created or analyzed in this study. Data sharing is not applicable to this article.

## Abbreviations

AHS	American Headache Society
CB	Carotid body
CGRP	Calcitonin gene-related peptide
CH	Cluster headache
CVLM	Caudal ventrolateral medulla
CYP2E1	Cytochrome P450-2E1
eNOS	Endothelial nitric oxide synthase
ER	Endoplasmic reticulum
ETC	Electron transport chain
GWAS	Genome-wide association study
HIF	Hypoxia-inducible factor
IL-1β	Interleukin-1 beta
IL-6	Interleukin-6
iNOS	Inducible nitric oxide synthase
JNK	c-Jun N-terminal kinase
MAPK	Mitogen-activated protein kinase
NF-κB	Nuclear factor-kappa B
NTS	Nucleus tractus solitarius
PACAP38	Pituitary adenylate cyclase-activating polypeptide-38
PHD	Prolyl hydrolase domain enzyme
pO_2_	Partial pressure of oxygen
PVN	Paraventricular nucleus of the hypothalamus
ROS	Reactive oxygen species
RVLM	Rostral ventrolateral medulla
SCN	Suprachiasmatic nucleus
SDH	Succinate dehydrogenase
TLR4	Toll-like receptor 4
TNC	Trigeminal nucleus caudalis
VIP	Vasoactive intestinal peptide
